# Comparative efficacy, safety and benefit/risk of alerting agents for excessive daytime sleepiness in patients with obstructive sleep apnoea: a network meta-analysis

**DOI:** 10.1016/j.eclinm.2024.102843

**Published:** 2024-09-19

**Authors:** Jean-Louis Pépin, Philippe Lehert, Raoua Ben Messaoud, Marie Joyeux-Faure, Christian Caussé, Jerryll Asin, Ferran Barbé, Maria R. Bonsignore, Winfried Randerath, Johan Verbraecken, Sonya Craig, Yves Dauvilliers

**Affiliations:** a Hypoxia-Physiopathology (HP2) Laboratory, INSERM U1300, University Grenoble Alpes, Grenoble, France; b Cardio-Respiratory Functional Exploration Laboratory (EFCR), Grenoble Alpes University Hospital, Grenoble, France; c Louvain School of Management, Louvain University, Chaussee de Binche 151/M1.01.01, 7000, Mons, Belgium; d Faculty of Medicine, The University of Melbourne, Parkville, VIC, Australia; e Bioprojet, Paris, France; f Center for Sleep Medicine, Department of Pulmonary Diseases, Amphia, Breda, the Netherlands; g Respiratory Dept, Institut Ricerca Biomedica de Vilanova, Lleida, Spain; h Sleep Disordered Breathing Clinic, Pulmonary Division, PROMISE Department, University of Palermo, Palermo, Italy; i National Research Council (CNR), Institute for Biomedical Research and Innovation (IRIB), Palermo, Italy; j Bethanien Hospital, Clinic of Pneumology and Allergology, Centre for Sleep Medicine and Respiratory Care, Institute of Pneumology at the University of Cologne, Solingen, Germany; k Multidisciplinary Sleep Disorders Centre, Antwerp University Hospital, Edegem, Belgium; l Laboratory of Experimental Medicine and Pediatrics (LEMP), Faculty of Medicine and Health Sciences, University of Antwerp, Wilrijk, Belgium; m Department of Respiratory Medicine, Aintree University Hospital, Liverpool University Hospitals NHS Foundation Trust, Liverpool, UK; n National Reference Center for Narcolepsy, Sleep and Wake Unit, Department of Neurology, Gui-de-Chauliac Hospital, Montpellier University Hospital, Montpellier, France; o INSERM U1061, Montpellier University, Montpellier, France

**Keywords:** Systematic review, Network meta-analysis, Obstructive sleep apnoea

## Abstract

**Background:**

Obstructive sleep apnoea (OSA) is a common chronic respiratory disease associated with a high burden of disabilities related to sleepiness and reduced quality of life. Despite first-line treatment with continuous positive airway pressure (CPAP) therapy, many patients experience residual excessive daytime sleepiness (EDS). The aim of this study is to compare the relative efficacy and safety of medications authorised for this indication in Europe and/or the United States (modafinil/armodafinil, solriamfetol, and pitolisant) for OSA.

**Methods:**

In this systematic review and network meta-analysis, randomised controlled trials (RCTs) that compared the efficacy and safety of authorised medications for adult patients with OSA were identified by literature searches of PubMed, Embase and ClinicalTrials.gov databases (up to 12 June 2024). The primary efficacy endpoint was combined Epworth Sleepiness Scale (ESS) and Oxford Sleep Resistance (OSLER)/Maintenance of Wakefulness Test (MWT) Z-scores. Quality of life (QoL), overall and specific cardiovascular safety, and benefit-risk ratios were calculated. The study was registered with PROSPERO: CRD42023434640.

**Findings:**

Of 4017 studies identified, a total of 20 RCTs involving 4015 patients were included. Analysis of combined subjective (ESS) and objective (OSLER/MWT) efficacy outcome Z-scores showed that solriamfetol (150 mg; effect size [ES] = 0.66 [95% CI: 0.36, 0.96]), pitolisant (20 mg; ES = 0.66 [95% CI: 0.44, 0.88]), and modafinil (200 mg; ES = 0.54: [95% CI: 0.33, 0.74]); 400 mg; ES = 0.54 [95% CI: 0.42, 0.65]) had a clinically meaningful improvement in efficacy. P-scores ranked placebo, then pitolisant, modafinil 200 mg, modafinil 400 mg and solriamfetol for overall safety; and pitolisant, then solriamfetol, modafinil 400 mg and modafinil 200 mg for benefit-risk ratio.

**Interpretation:**

Pitolisant, solriamfetol and modafinil had comparable efficacy for maintaining wakefulness in patients with OSA. Pitolisant had a better safety profile and benefit-risk ratio compared with solriamfetol and modafinil. The overall and cardiovascular safety risk ratios suggest that pitolisant might be the best candidate for patients with OSA with multiple cardiovascular comorbidities.

**Funding:**

10.13039/100030995Bioprojet.

**Research in context**

**Evidence before this study**

Previous meta-analyses for obstructive sleep apnoea (OSA) have analysed studies of single pharmacological agents *versus* placebo. Network meta-analysis is a tool developed to overcome some limitations of pairwise meta-analyses. In this systematic review and network meta-analysis, randomised controlled trials (RCTs) that compared the efficacy and safety of authorised medications for adult patients with OSA were identified by literature searches of PubMed, Embase and ClinicalTrials.gov databases (up to 12 June 2024).

**Added value of this study**

This network meta-analysis analysed the largest number of RCTs (n = 20) of OSA published prior to data cutoff and included only data from authorised drugs (modafinil/armodafinil, solriamfetol, and pitolisant) and comparators (mainly placebo). The study provided Z-scores for: efficacy by combining subjective and objective outcomes, quality of life, overall and cardiovascular safety (blood pressure and heart rate) and benefit-risk, as well as univariate efficacy and safety variables.

**Implications of all the available evidence**

Pitolisant, solriamfetol and modafinil had comparable efficacy for maintaining wakefulness/vigilance in patients with OSA. Pitolisant might be the best candidate for patients with OSA with multiple comorbidities such as cardiovascular disorders linked to a better safety and cardiovascular profile and benefit-risk ratio compared with solriamfetol and modafinil. Longer-term RCTs which prioritise head-to-head comparisons of these medications are needed.

## Introduction

Obstructive sleep apnoea (OSA) is a common clinical condition with partial or complete pharyngeal collapses occurring repeatedly during sleep, causing apnoeas and hypopnoeas leading to intermittent hypoxia and sleep disruption.^1^^,^^2^ Nearly 1 billion people are affected globally^3^ and the disease is associated with a high prevalence of cardiometabolic comorbidities impacting prognosis.^1^ OSA is associated with a substantial economic societal burden,^4^ with excessive daytime sleepiness (EDS), being a recognised risk factor for motor vehicle accidents.^5^ EDS may remain despite first-line treatment with continuous positive airway pressure (CPAP) therapy.^2^^,^^6^^,^^7^ Approximately 13% of patients treated with CPAP experience residual daytime sleepiness, with a complex and multiple pathogenesis that is often associated with comorbid conditions such as insufficient sleep and depression.^8^ EDS can be evaluated using the subjective Epworth Sleepiness Scale (ESS) or with objective tests such as the Maintenance of Wakefulness Test (MWT) and the Oxford Sleep Resistance (OSLER) test.^7^^,^^9^

Several pharmacologic treatments for OSA-related EDS are available. Randomised controlled trials (RCTs) have shown that modafinil and its R-enantiomer, armodafinil,^10^, ^11^, ^12^, ^13^, ^14^, ^15^, ^16^, ^17^, ^18^, ^19^, ^20^, ^21^, ^22^, ^23^, ^24^ solriamfetol,^25^, ^26^, ^27^, ^28^ and pitolisant^29^^,^^30^ are effective in improving EDS. Modafinil, armodafinil, and solriamfetol are approved in the United States for the treatment of EDS in adults with OSA. Regulatory approval for modafinil/armodafinil for this indication was withdrawn by the European Medicines Agency (EMA) in 2011 due to safety concerns. Both solriamfetol and pitolisant are approved in the EU for patients with OSA whose EDS has not been treated satisfactorily by primary OSA therapy (e.g. CPAP).^2^^,^^7^^,^^9^

Drugs available for treating OSA-associated EDS have only been compared in RCTs against placebo and there have been no direct comparisons of drugs approved for OSA. One way to address this issue is to conduct a network meta-analysis to investigate the comparative effectiveness and safety of medications for OSA-related EDS.

Previous network meta-analyses comparing medications for EDS in OSA patients analysed 6^31^ and 14^32^ RCTs. The latter study assessed a limited number of outcomes and safety parameters and included data for non-commercially available drugs worldwide.^32^

The aim of this network meta-analysis is to compare the efficacy and safety of drugs approved in Europe or the United States for OSA therapy i.e. modafinil/armodafinil, solriamfetol, and pitolisant, and to test a larger number of efficacy and safety outcomes to reflect the benefit-risk ratio.

## Methods

The study protocol was registered (PROSPERO: CRD42023434640) and data locked on 12 June 2024. The revised PRISMA 2020 guidelines for systematic reviews were followed.^33^

### Study selection

Selection criteria were defined as the set of all RCTs published up to 12 June 2024 in adults (aged 18 years or older), diagnosed with OSA by validated sleep tests (polygraphy or polysomnography), and treated for EDS with a pharmacological agent (modafinil/armodafinil, pitolisant, or solriamfetol).

We used the search strategy adapted from SIGN guidance (https://www.sign.ac.uk/what-we-do/methodology/search-filters/) which recommends search terms for systematic reviews and meta-analyses: ((“Sleep apnoea, obstructive” [MeSH Terms] OR obstructive sleep apnoea) AND (“Sleep Apnoea Syndromes/Modafinil/Armodafinil ” [Mesh]) AND (“Sleep Apnoea Syndromes/Pitolisant” [Mesh]) AND (“Sleep Apnoea Syndromes/Solriamfetol” [Mesh])).

Searches of PubMed, Embase, and ClinicalTrials.gov databases were conducted, and manufacturers were also contacted for details of unpublished RCTs. All reference lists of selected studies and systematic reviews were checked to identify additional eligible studies. There was no restriction for language or the publication period for searching. The authors independently searched for potential studies and disagreement was solved by consensus.

### Participants

All randomised patients constituting the Intention-To-Treat population (i.e. Full Analysis Set [FAS]), were analysed.

### Interventions and comparators

All pharmacological treatments for EDS in OSA patients and approved in Europe and/or the United States were compared. Dosages analysed were: modafinil 200 mg and 400 mg, solriamfetol 150 mg, and pitolisant 20 mg. The 200 mg and 400 mg dosages of modafinil were considered to be equivalent to armodafinil 150 mg and 250 mg, respectively.^34^ Results for modafinil 100 mg were assimilated with those for modafinil 200 mg, as they were shown to be very similar. In a titration series, only the highest dose recommended for treatment was used. Placebo was the comparator in all selected RCTs. Solriamfetol 300 mg was not considered as it is not approved by regulatory agencies.

### Data collection

Data were collected by Raoua Ben Messaoud [RBM] and disputes were resolved by two additional reviewers, Philippe Lehert [PL] and Jean-Louis Pépin [JLP] who independently assessed and validated the findings.

#### Efficacy outcomes

ESS is a validated patient-reported outcome, self-administered questionnaire for assessing subjectively EDS severity in OSA patients. Responses are summed to yield a score between 0 and 24, with higher scores representing greater sleepiness. A score ≥11 is considered to indicate EDS.^35^

Objective outcomes used to provide a quantitative measurement of wakefulness were MWT, a polysomnographic technique which measures the maintenance of wakefulness^36^^,^^37^ and the OSLER test, which is a behavioural test for assessing daytime vigilance that estimates the maintenance of wakefulness.^38^

The main efficacy endpoint was a composite endpoint of ESS and OSLER/MWT and calculated as the mean of the sum of ESS and OSLER/MWT Z-scores.

Measures of quality of life (QoL) evaluated for sleepiness were the Functional Outcomes of Sleep Questionnaire (FOSQ),^39^ EuroQol five-dimension quality of life scale (EQ-5D),^40^ 36-item Short-Form health survey (SF-36),^41^ or the Pittsburgh Sleep Quality Index (PSQI).^42^

#### Safety outcomes

Pre-determined safety variables were systolic blood pressure (SBP), diastolic blood pressure (DBP) and assumed to be office blood pressure measurements (where not otherwise indicated), and heart rate (HR), treatment-emergent adverse events (TEAEs), and adverse events (AEs) of special interest, notably headache due to its high frequency in this population.

The overall safety endpoint was a composite endpoint based on the overall sum of the Z-scores of published safety endpoints.

### Statistics

#### Quality of evidence and risk of bias

Quality of evidence (high, moderate, low, and very low) was classified using the GRADE system.^43^ Risk of bias within each study was evaluated using the Cochrane, RoB 2 tool.^44^

#### Data analysis: calculations and transformations

A more detailed description of data transformations can be found in Supplementary Methods including: conversion of statistical parameters (mean, median, standard deviation [SD], standard error [SE], interquartiles, confidence interval [CI]); and assimilated mean change and final values based on a correlation baseline–final correlation R = 0.5; conversion of quantitative measures (standardized mean difference, mean change) into binary values (risk or odds ratio) and vice-versa; summary of the same endpoint in the same study assuming their correlations approximated by compound symmetry matrix; composite endpoints based on different endpoints (such as safety), based on the sum of their Z-scores weight by using a correlation matrix following the Mahalanobis metric. Both QoL measures and safety outcomes were weighted using the Mahalanobis metric. R software (release 4.3.3)^45^ and the netmeta library^46^ were used for the meta-analysis.

#### Benefit-risk evaluation

The benefit-risk evaluation of each treatment was estimated according to a compensatory additive model based on the efficacy and safety overall scores, equally weighted according to the Fishbein-Ajzen model^47^ as applied by Bouyssou.^48^ For this, we assumed that efficacy and safety variables are opposites; thus, the overall sum estimating the benefit-risk is the sum of positive efficacy and negative safety values. Principal components analysis (PCA) was used to graphically represent the characteristics of the studied treatments using a vectorial model.^49^

#### Multiple testing

To control for multiple testing, a two-sided significance level of 0.025 for aggregated efficacy and safety Z-scores was adopted. Significance for benefit-risk was considered when significance level was achieved for either efficacy or safety Z-scores.

#### Statistical modelling

Network meta-analysis was performed using the Rücker and Schwarzer method.^50^

Network inconsistency and heterogeneity was calculated using Cochran's Q test. Qt (overall difference between observed values and the network estimates), Qh (heterogeneity between studies) and Qi (inconsistency between designs and transitivity) were tested with χ^2^ tests. Inferential methods were used to estimate the effect size (with 95% CI) of pairwise comparisons, and the corresponding *p* value of effect size differences. Treatments were ranked by calculating P-scores.^51^ Pi is defined as the mean of all 1–Pj (Pj = one-sided *p* value of accepting the alternative hypothesis that ti > tj). Pi can be interpreted as the mean extent of certainty that treatment *i* is better than another treatment and is comparable to that of the Surface Under the Cumulative Ranking curve (SUCRA) which is the rank of treatment *i* within the range of treatments and measured on a scale from 0 (worst) to 1 (best).^52^

### Role of funding source

Representatives of the study sponsor were involved in the study design, collection, analysis and interpretation of data, writing of the report, and in the decision to submit the paper for publication. The first draft of the manuscript was prepared by JLP, PL and YD. Medical writing assistance was provided by an independent medical writer funded by Bioprojet. The manuscript was reviewed and edited by all the authors. All authors made the decision to submit the manuscript for publication and assume responsibility for the accuracy and completeness of the analyses and for the fidelity of this report to the study protocol.

## Results

**Fig. 1:**
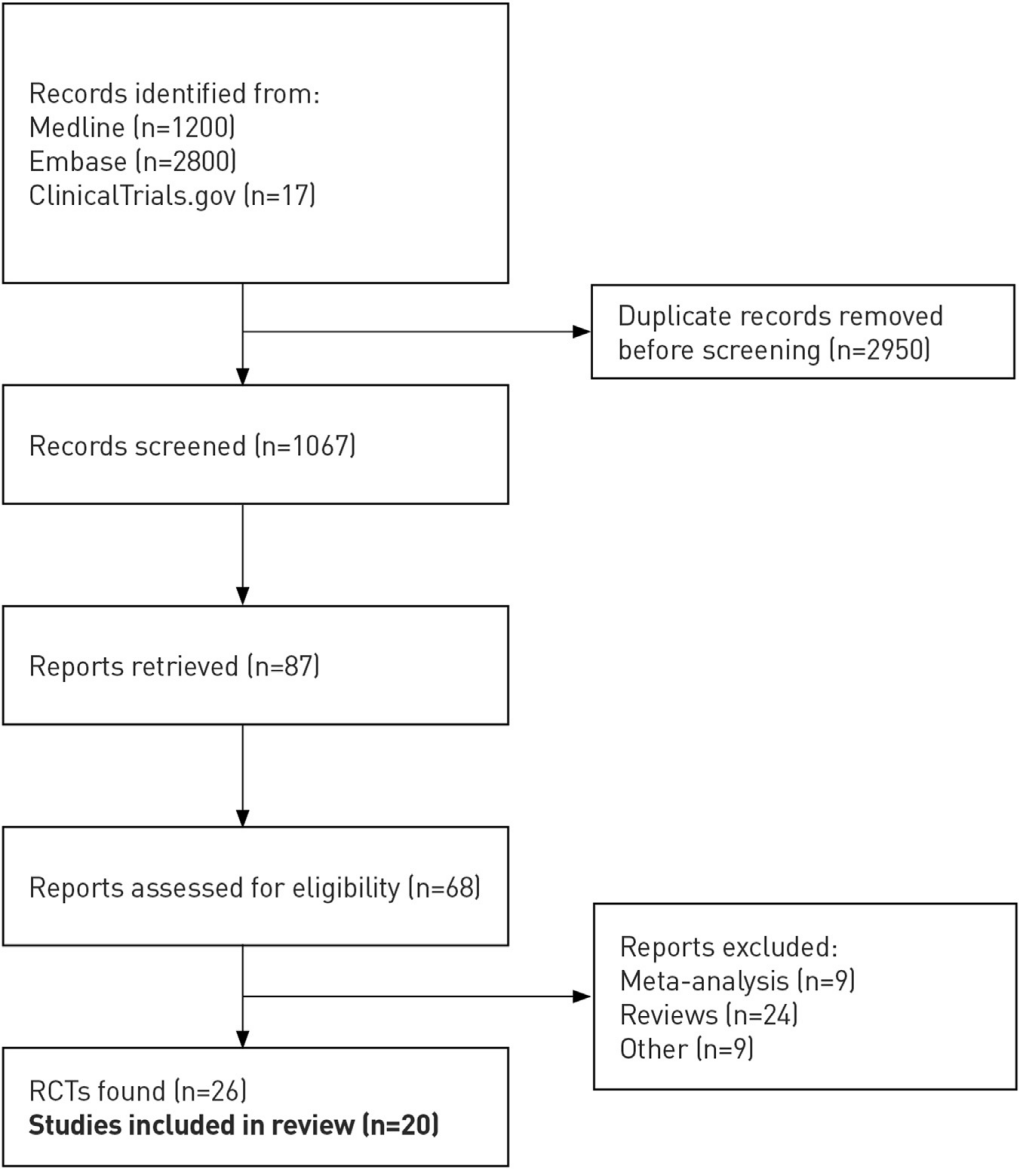
**Study selection**.

Of 4017 records identified, a total of 26 RCTs were eligible (Fig. 1). Six RCTs were excluded: (1) Kay et al. (2013) which studied the effects of armodafinil on simulated driving^53^; (2) Arnulf et al. (1997): a pilot study limited to 6 cases^54^; (3) Schartz et al., 2003: an open-label study of modafinil^55^; (4) Malhotra et al. (2020): a long-term study of solriamfetol maintenance therapy^56^; (5) Schweitzer et al. (2021): an open-label extension trial of solriamfetol which compared adherent *versus* non-adherent patients^57^; and (6) Williams et al. (2008) which studied the effect of modafinil on neurobehavioural performance in a crossover trial of 12 patients.^58^ Twenty RCTs were included in the network meta-analysis. Of these, the Strollo et al. (2019) trial was evaluated for safety but not for efficacy analyses as responders were randomly assigned to placebo or solriamfetol at week 4, for 2 weeks only.^27^ In the Herring et al. (2013) study,^15^ data for the histamine-3 inverse agonist MK-0249 were excluded as it is not commercially available, but data for modafinil 200 mg and placebo were analysed. Apart from three crossover trials,^12^^,^^15^^,^^19^ RCTs had a parallel group design (Table 1).

**Table 1:** Characteristics of studies (n = 20) included in the network meta-analysis.

Study	Trial registration	Study design	Randomised (n)	Countries	Male (%)	Mean				Follow up (weeks)	Main endpoints	Number of reported endpoints per tested treatment				
						Age (y)	BMI (kg/m^2^)	ESS	AHI (events/h)							
												MDF2	MDF4	PITL	SMFT	PLA
Bittencourt et al. 2008^10^	NR	RCT, db	22	Brazil	85	53	33.5	NR	NR	4	ESS, MWT, CGI-C, SF-36	4	0	0	0	4
Black et al. 2005^11^	NR	RCT, db, PG	309	United states, United Kingdom	75.7	49.3	36.8	NR	4.7	12	MWT, ESS, FOSQ, CGI-C, AEs	11	11	0	0	11
Chapman et al. 2014^12^	ACTRN#12608000128392	RCT, CT	32	Australia	13.7	47	28.2	12.5	15.2	12	ESS, FOSQ, AEs	4	0	0	0	4
Dauvilliers et al. 2020^29^	NCT01072968	RCT, db, PG	268	France	75.4	52	32.9	15.7	NR	12	ESS, OSLER, CGI-S, PGO, EQ-5D, PFQ, safety	0	0	13	0	13
Dinges et al. 2003^13^	NR	RCT, db, PG	157	United states	76.5	50	35.5	14.3	NR	4	PVT, FOSQ	0	6	0	0	6
Greve et al. 2014^14^	NCT00711516	RCT, db	40	United States	77.7	50.3	32.8	15.6	NR	2	CANTAB, AEs	0	1	0	0	1
Herring et al. 2013^15^	NCT00620659	RCT, db, CT	125	United States	80	48.6	NR	15.3	NR	2	MWT, PVT, ESS, CGI-S, DSST, AEs	0	2	0	0	2
Hirshkowitz et al. 2007^16^	NR	RCT, db	263	United States, Australia, Germany, France	73.5	50.6	33.5	15.8	1.3	12	MWT, ESS, CGI-C, CDR, BFI, AEs	0	2^a^	0	0	2
Inoue et al. 2013^18^	JapicCTI-No. 090777	RCT, db	114	Japan	96.5	49.8	27.6	14.5	2.7	4	ESS, MWT, PSQI, Safety	4	0	0	0	0
Inoue et al. 2016^17^	AFT-801-0305	RCT, db, PG	50	Japan	94	52	27.3	14.1	3	4	ESS, MWT	2	0	0	0	0
Kingshott et al. 2001^19^	NR	RCT, db, CT	30	United Kingdom	NR	53	32	15	8	3	ESS, MWT, MSLT, SF-36, FOSQ, AEs	0	11	0	0	11
Krystal et al. 2010^20^	NCT00518986	RCT, db	248	United States	46.5	49.5	36.8	14.8		12	MWT, CGI-C, ESS, AEs	0	2^a^	0	0	2
Pack et al. 2001^21^	NR	RCT, db, PG	157	United States	74	50	35.5	14.3	5.8	4	ESS, MSLT, AEs	0	4	0	0	0
Pépin et al. 2021^30^	NCT01071876; EudraCT 2009-017251-94	RCT, db, PG	244	France	82.8	53.1	32.6	14.7	4.2	12	ESS, OSLER, CGI-S, PGO, EQ-5D, PFQ, Safety	0	0	13	0	13
Roth et al. 2006^22^	NR	RCT, db	395	United States, Canada	70.4	49.5	36.7	15.5	1.5	12	MWT, CGI-C, ESS, BFI, AEs	0	2^a^	0	0	2
Schweitzer et al. 2019^26^	NCT02348606; EudraCT 2014-005514-31	RCT, db, PG	476	United States, Canada, France, Germany, Netherlands	62.6	53.9	33.3	15.2	NR	12	SL, MWT, ESS, PGI-C, AEs	0	0	0	10	0
Strollo et al. 2019^27^	NCT02348619; EudraCT 2014-005515-16	RCT	124	Finland, France Germany, Sweden, United States	61.8	55.4	33.3	NR	NR	4	MWT, ESS, AEs	0	0	0	10	10
Weaver et al. 2009^23^	NR	RCT, db	480	United States	78	49.7		15.2	NR	12	FOSQ	0	6	0	0	0
Weaver et al. 2020^28^	NCT02348606	RCT	459	United States, Canada, France, Germany, Netherlands	62.5	54.3	33.4	15.2	NR	12	FOSQ, SF-36, WPAIQ, Safety	0	0	0	6	6
Williams et al. 2010^24^	ACTRN12606000027516	RCT, db	21	Australia	100	55	32.7	8.7	2.6	12	AusEd driving simulator, PVT, KSS	1	0	0	0	0

AEs, adverse events; BFI, Brief Fatigue Inventory; CANTAB, Cambridge Neuropsychological Test Automated Battery; CDR, Cognitive Drug Research battery; CGI, Clinical Global Impression; CGI-C, Clinical Global Impression of Change; CGI-S, Clinical Global Impression of Severity; CPAP, continuous positive airway pressure; CT, crossover trial; db, double-blind; DSS, Driving Safety Score; DSST, Digit Symbol Substitution Test; EQ-5D, EuroQoL five-dimension quality-of-life questionnaire; ESS, Epworth Sleepiness Scale; FOSQ, Functional Outcomes of Sleep Questionnaire; KSS, Karolinska Sleepiness Scale; MSLT, Multiple Sleep Latency Test; MWT, maintenance of wakefulness test; OSLER, Oxford Sleep Resistance test; PFQ, Pichot's Fatigue Questionnaire; PG, parallel group; PGI-C, Patient Global Impression of Change; PGO, Patient's Global Opinion; PSQI, Pittsburgh Sleep Quality Index; PVT, Psychomotor Vigilance Task; NR, not reported; RCT, randomized controlled trial; SF-36, 36-Item Short Form Survey; SL, sleep latency; WPAIQ, Work Productivity and Activity Impairment Questionnaire.

a Modafinil-equivalent doses of armodafinil; MDF2, modafinil 200 mg; MDF4, modafinil 400 mg; PITL, pitolisant; SMFT, solriamfetol; PLA, placebo.

Most studies were conducted in Europe and the USA, and most patients were Caucasian. All trial participants had an ESS score at baseline of at least 10. Almost all trials used conventional CPAP or mandibular device OSA therapy, except Dauvilliers et al. (2020).^29^ All the trials did not include patients with severe or unstable disease other than OSA, especially unstable comorbid cardiovascular disease.

A total of 4015 patients from 20 RCTs were included in the meta-analysis. At baseline, patients had a mean age of 51.6 years (range across studies 47–55 years) and 69.5% were male. Patients had a mean (SD) BMI of 33.2 (2.38) kg/m^2^ and mean (SD) ESS of 14.5 (1.73). The most common trial duration was 12 weeks (9/20 RCTs) (Table 1).

Risk of bias in selected RCTs was assessed to be low or probably low (Supplementary Table S1).

**Fig. 2:**
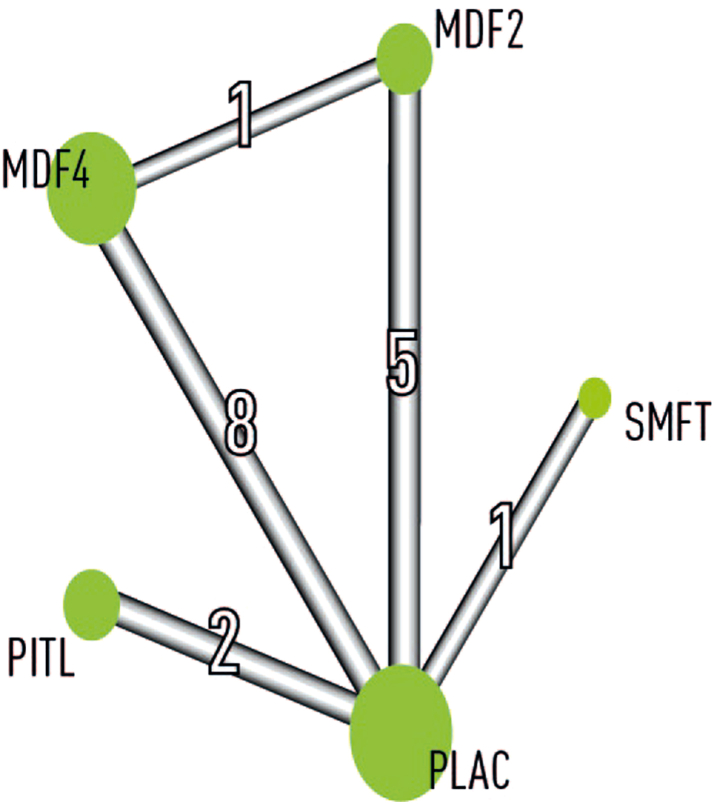
**Network diagram for efficacy outcomes**. The thickness of lines (edges) joining two treatments is proportional to the inverse standard error of the comparison between these two treatments. The number in the middle of each edge is the number of studies used for estimating the effect between the two joined treatments. Each treatment is represented by a green circle with a radius that is proportional to the number of the patients having received this treatment. MDF2, modafinil 200 mg; MDF4, modafinil 400 mg; PITL, pitolisant; PLAC, placebo; SMFT, solriamfetol.

The network diagram for efficacy outcomes is shown in Fig. 2. Of a total number of 4015 patients, the largest number of patients in direct pairwise comparisons involved placebo (1498), followed by modafinil 400 mg (1056), pitolisant (383) and modafinil 200 mg (188), and solriamfetol (116).

**Fig. 3:**
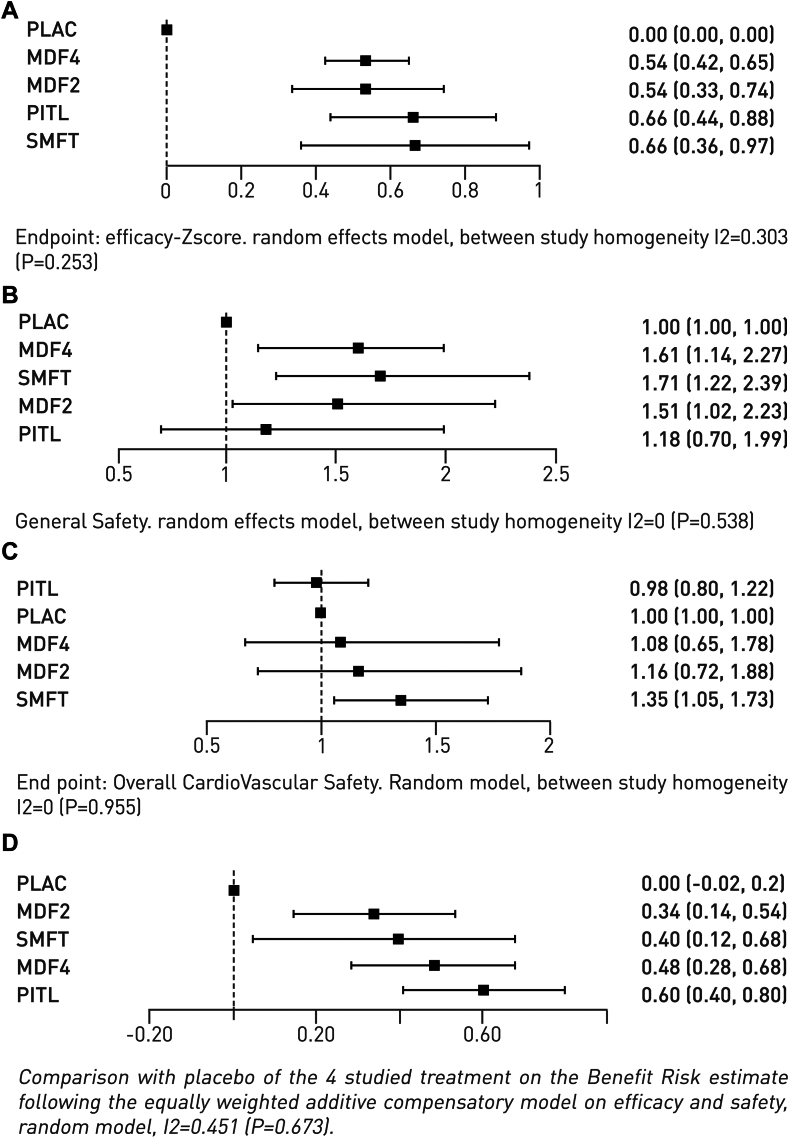
**A. Forest plot for efficacy Z-score (ESS + OSLER/MWT)**. Random Effects Model, effect sizes (95% CI) relative to placebo are shown. MDF2, modafinil 200 mg; MDF4, modafinil 400 mg; PITL, pitolisant; SMFT, solriamfetol. **B. Forest plot for overall safety (risk ratio)**. Random Effects Model, effect sizes (95% CI) relative to placebo are shown. Comparison of Risk Ratios of at least one Treatment-Emergent Adverse Event (TEAE) of any type. MDF2, modafinil 200 mg; MDF4, modafinil 400 mg; PITL, pitolisant; SMFT, solriamfetol. **C. Forest plot for cardiovascular safety (risk ratio)**. Random Effects Model, effect sizes (95% CI) relative to placebo are shown. MDF2, modafinil 200 mg; MDF4, modafinil 400 mg; PITL, pitolisant; SMFT, solriamfetol. **D. Forest plot for benefit/risk BR ratio**. Random Effects Model, effect sizes (95% CI) relative to placebo are shown. MDF2, modafinil 200 mg; MDF4, modafinil 400 mg; PITL, pitolisant; SMFT, solriamfetol.

Analysis of the primary efficacy endpoint of combined ESS and OSLER/MWT Z-scores included 12 RCTs which showed low-moderate heterogeneity (I^2^ = 28.9%; P = 0.253). Each of the four comparators had a significant treatment effect compared with the placebo. Fig. 3A shows a Forest plot for efficacy Z-scores. Pitolisant, solriamfetol, and modafinil (both doses) each had a clinically meaningful improvement in efficacy as the effect size (mean standardized mean difference) was >0.2.^59^ Pitolisant and solriamfetol had very similar P-scores for the combined efficacy endpoint (Table 2).

**Table 2:** P-scores for comparison between treatments for efficacy, benefit-risk and quality of life (QoL). Drugs are ranked by P-score for each endpoint.

Endpoint	Placebo	Modafinil 200 mg	Modafinil 400 mg	Pitolisant	Solriamfetol
Efficacy Z-score (ESS + OSLER/MWT)	**0.00**	*0.48*	*0.47*	*0.78*	*0.76*
Ranking	5	3	4	1	2
ESS	**0.01**	*0.57*	*0.57*	*0.63*	*0.73*
Ranking	5	3	3	2	1
OSLER/MWT	**0.03**	0.51	*0.62*	*0.66*	*0.69*
Ranking	5	4	3	2	1
Benefit-risk (efficacy/safety)	**0.00**	0.32	*0.56*	*0.81*	*0.67*
Ranking	5	4	3	1	2
QoL	0.27	0.25	0.69	0.73	0.66
Ranking	4	5	2	1	3

For each parameter, a P-score highlighted in *yellow* signifies a significant one-sided benefit of a treatment compared with a **blue**-highlighted treatment: a P-score closer to 1 indicates relative superiority.

ESS, Epworth Sleepiness Scale; MWT, maintenance of wakefulness test; OSLER, Oxford Sleep Resistance test, QoL, quality of life.

Forest plots for analysis of ESS and OSLER/MWT alone are shown in Supplementary Figures S1 and S2, respectively. Heterogeneity between studies was high for both ESS (I^2^ = 82.2%) and OSLER/MWT (I^2^ = 78.6%). P-scores showed significant benefits of pitolisant, solriamfetol, and modafinil (both doses) for ESS improvement and of pitolisant, solriamfetol, and modafinil 400 mg for improving OSLER/MWT scores (Table 2). There were no significant differences between the treatments for QoL although heterogeneity between studies was high (I^2^ = 81.2%) (Supplementary Figure S3; Table 2).

Safety analysis was conducted on a total sample of 2208 patients in 12 RCTs with most involving placebo (881), then modafinil 400 mg (385), pitolisant (384), solriamfetol (290), and modafinil 200 mg (267). The network diagram for safety is shown in Supplementary Figure S4. Fig. 3B shows a Forest plot for the composite overall safety score based on risk ratios. Solriamfetol and modafinil (both doses) had effect sizes ≥0.2. Overall safety by P-scores ranked placebo, then pitolisant, modafinil 200 mg, modafinil 400 mg, and solriamfetol (Table 3). Fig. 3C shows a Forest plot for cardiovascular safety based on risk ratios. Order of ranking by P-scores was pitolisant, then placebo, modafinil 400 mg, modafinil 200 mg, and solriamfetol (Table 3). Forest plots for overall and cardiovascular safety based on Z-scores are shown in Supplementary Figures S5 and S6, respectively. Study heterogeneity was low for all overall and cardiovascular safety analyses (I^2^ = 0%). Modafinil (both doses) and solriamfetol had significantly lower safety P-scores than placebo for both overall and cardiovascular safety irrespective of the model used. Forest plots for safety analyses including SBP, DBP, HR, TEAEs, headache, TEAEs (except headache), and SAEs are shown in Supplementary Figures S7–S13. P-scores for placebo and pitolisant were comparable for all these safety parameters (Table 3).

**Table 3:** P-scores for comparison between treatments for safety parameters.

Parameter	Placebo	Modafinil 200 mg	Modafinil 400 mg	Pitolisant	Solriamfetol
Overall safety (Risk ratio)	*0.93*	**0.39**	**0.29**	0.69	**0.21**
Ranking	1	3	4	2	5
Overall safety (Z-score)	*0.91*	**0.37**	**0.29**	0.81	**0.12**
Ranking	1	3	4	2	5
Cardiovascular safety (Risk ratio)	*0.7*	**0.42**	**0.53**	0.72	**0.14**
Ranking	2	4	3	1	5
Cardiovascular safety (Z-score)	*0.67*	**0.4**	**0.51**	0.8	**0.13**
Ranking	2	4	3	1	5
All TEAEs	*0.94*	**0.47**	**0.31**	0.56	**0.22**
Ranking	1	3	4	2	5
SAEs	0.8	NA	0.35	0.52	0.32
Ranking	1	–	3	2	4
Headache	*0.83*	0.31	**0.29**	0.81	**0.25**
Ranking	1	4	3	2	5
TEAEs (not headache)	*0.91*	0.68	**0.13**	0.35	0.43
Ranking	1	2	5	4	3
Systolic blood pressure	0.67	0.3	0.49	0.74	0.3
Ranking	2	4	3	1	4
Diastolic blood pressure	0.59	0.5	0.4	0.78	0.23
Ranking	2	3	4	1	5
Heart rate	*0.73*	NA	NA	0.75	**0.02**
Ranking	2	–	–	1	3

For each parameter, a P-score highlighted in *yellow* signifies a significant one-sided benefit of a treatment compared with a **blue**-highlighted treatment: a P-score closer to 1 indicates relative superiority.

SAE, serious adverse event; TEAE, treatment emergent adverse event.

Drugs are ranked by P-score for each parameter.

A Forest plot for benefit-risk, indicates that each treatment had a benefit-risk ratio superior to placebo. Solriamfetol and modafinil (both doses) had effect sizes >0.2 indicating a small Minimal Clinically Important Difference (MCID) in benefit-risk,^59^ whilst pitolisant had a medium MCID (effect size >0.5) (Fig. 3D). P-scores ranked pitolisant, then solriamfetol, modafinil 400 mg and modafinil 200 mg for benefit-risk (Table 2).

**Fig. 4:**
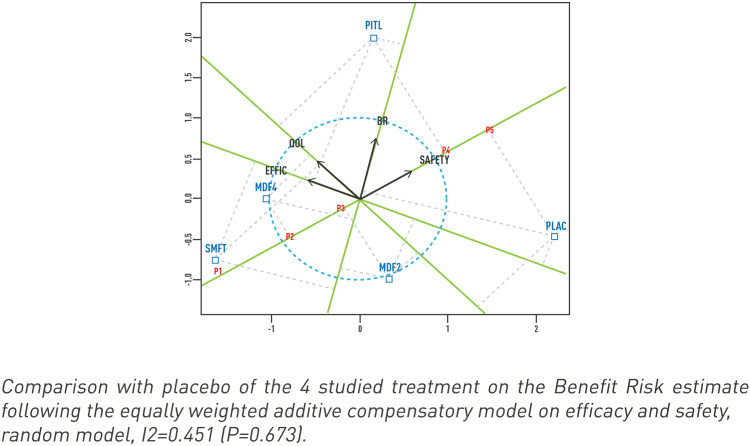
**Principal components analysis (PCA) of each treatment relative to efficacy (EFFIC), safety, benefit-risk (BR) and quality of life (QOL) axes (green lines)**. This is a summarized graphical comparison of treatments on multiple possibly discordant criteria in showing the treatments and the characteristics (efficacy, safety, QoL benefit-risk) on the two main factors F1 (Explained Variance [EV] = 48%) and F2 (EV = 34%) of a principal components analysis (PCA). The figure allows various conclusions: (1) Each characteristic (Efficacy, Safety, Benefit-risk and Quality of life) is represented by a green directed axis. The angle between two variables visualizes their correlation, axes with opposed directions are inversely correlated: QoL and efficacy are correlated, whereas safety and efficacy are inversely correlated. (2) The projections of each treatment on an axis are an approximate measure of the treatment on this axis, and the axis origin is the average for all the measurements. For instance, looking at safety by decreasing value, the best treatment is placebo (P5 point) followed by pitolisant (P4), etc. until solriamfetol (P1) whereas, for efficacy, the projections are in reverse order. (3) The mutual position of the treatments (blue points) suggests a visual clustering: placebo alone with an obvious image of good safety combined with a lack of efficacy; the two treatments SMFT and MDF4 characterized by good efficacy but less satisfactory safety, MDF2 assimilable with this group. Finally, pitolisant constitutes a third cluster characterized by both satisfactory efficacy and safety resulting in an optimal benefit-risk value. QoL appears to be more correlated with efficacy, and SMFT, MDF4 and PITL are shown to be similar on this dimension. MDF2, modafinil 200 mg; MDF4, modafinil 400 mg; PITL, pitolisant; PLAC, placebo; SMFT, solriamfetol. Projections of each treatment on the safety axis are shown for placebo (P5), pitolisant (P4), modafinil 200 mg (P3), modafinil 400 mg (P2), and solriamfetol (P1).

PCA of each treatment relative to efficacy, safety, benefit-risk and QoL axes is shown in Fig. 4. For safety, the best treatment was pitolisant, then modafinil 200 mg, modafinil 400 mg and solriamfetol. The projections of modafinil (200 mg and 400 mg) and solriamfetol on the Safety axis lie below the average point of all the measurements (centre of the graphic, where axes converge). For efficacy, the best treatment was solriamfetol followed by a group comprising modafinil 400 mg and pitolisant, then modafinil 200 mg, and finally placebo. Four clusters are evident and defined by placebo (good safety, poor efficacy); solriamfetol and modafinil 400 mg (good efficacy, moderate safety), modafinil 200 mg (moderate efficacy and safety) and pitolisant (good efficacy and safety) resulting in an optimal benefit-risk for pitolisant.

## Discussion

This network meta-analysis of 20 RCTs which included 4015 adult patients with OSA showed that pitolisant, solriamfetol and modafinil had comparable efficacy for maintaining wakefulness as assessed using a composite endpoint of mean Z-scores for subjective (ESS) and objective (OSLER/MWT) outcomes. In the present meta-analysis, pitolisant had a better safety profile and benefit-risk ratio compared with solriamfetol and modafinil. The overall cardiovascular safety results suggest that pitolisant might be the best candidate for patients with OSA with multiple comorbidities, especially with CVD.

Previous meta-analyses for EDS included studies limited to comparisons of modafinil with placebo in OSA^60^^,^^61^; network meta-analyses of studies comparing pharmacological interventions in narcolepsy^62^^,^^63^; comparison of pitolisant *versus* placebo in patients with OSA only^64^ or in patients with narcolepsy or OSA^65^; studies of solriamfetol *versus* placebo in patients with narcolepsy/OSA^66^; and network meta-analyses comparing medications for EDS in OSA.^33^^,^^34^ Previous network meta-analyses of OSA analysed 1714 participants in 6 RCTs^33^ and 3085 patients in 14 RCTs.^34^ In their network meta-analysis, Pitre et al. (2023) also showed that solriamfetol, modafinil-armodafinil, and pitolisant reduce daytime sleepiness for patients with OSA receiving conventional therapy, but their results indicated that solriamfetol was likely to be superior although they did not include OSLER tests documenting pitolisant improvements in objective assessment. In agreement with the current study, the safety profile of pitolisant was superior to that of solriamfetol and modafinil/armodafinil.^34^ This safety analysis, including cardiovascular safety in the current work, is of particular importance for guiding clinical decisions in the multimorbid population with OSA.

Our larger meta-analysis compared medications which are approved in Europe and/or the United States at doses commonly used in RCTs (rather than aggregating all dosages). We consider that this approach is closer to routine clinical practice, where dose-effect is significant. This is important as the EMA and U.S. Food and Drug Administration (*FDA*) recommended dose for solriamfetol is 150 mg and the FDA recommended dose for modafinil is 200 mg in OSA. In contrast to the meta-analysis of Pitre et al. (2023),^34^ we excluded efficacy data from Strollo et al., 2019 as patient responders were randomly assigned to placebo or solriamfetol for 2 weeks only, and data for the histamine-3 inverse agonist MK-0249 which is not commercially available.^27^ We used two composite endpoints (ESS and OSLER/MWT) to control for multiplicity of testing but also confirmed our results using separate univariate endpoints and combined all safety data and cardiovascular safety data as well as analysing individual safety parameters including SBP, DBP and HR, and headache, due to its prevalence in RCTs. Finally, we estimated benefit-risk based on a compensatory model for efficacy and safety and assessed risk-utility which showed superiority of pitolisant compared with solriamfetol and modafinil.

Limitations of this study include the lack of consistent outcomes for patients with OSA. Consequently, we used a composite endpoint of mean Z-scores for subjective and objective outcomes which may limit interpretability for clinicians. Setting the effect size to 0.2, indicating a small clinically meaningful improvement in efficacy,^59^ is arbitrary and should not be interpreted rigidly.^67^ As there is a lack of connectivity of our network, assumptions regarding transitivity and coherence are difficult to assess. The clinical characteristics of most participants in the selected RCTs limit the generalizability of the results: approximately 70% were men, were mainly white and were receiving conventional therapy. Furthermore, patient inclusion criteria were variable e.g. baseline ESS varied from ≥10 to ≥12, and adherence/non-adherence to CPAP therapy. Most trials were conducted in high-income countries in Europe, Japan, the USA or Australia, and the most common trial duration was 12 weeks (9/20 RCTs) with others conducted from 2 to 6 weeks. Study design of selected trials differed: most had a parallel group design but three crossover RCTs were included, and trials differed in fixed *versus* escalating dose of investigational product. Efficacy for solriamfetol was based on a single RCT,^26^ and trials of both modafinil and armodafinil were included with dosages of armodafinil converted to `modafinil-equivalent doses based on a head-to-head clinical trial.^36^ The latter approach (combining modafinil and armodafinil data) did not result in any significant changes to the results of our analysis compared with a repeated analysis of the two drugs separately for the main endpoints (ESS, MWT and Z-scores; data not shown). The objective assessment of EDS was carried out with the OSLER for both pitolisant RCTs,^29^^,^^30^ whereas MWT was the preferred instrument tool for modafinil and solriamfetol trials (the co-primary endpoints in the solriamfetol RCT were ESS and MWT,^26^ whereas the primary endpoint in pitolisant RCTs was ESS, and OSLER was a secondary outcome^29^^,^^30^). Using the standardized mean difference allowed us to avoid the expected heterogeneity on the mean and standard deviation of the two measures, and the choice of a random meta-analytical model attempted to capture an induced possible study effect. Analysis of different instruments for QoL may have contributed to the high degree of heterogeneity between studies. Furthermore, all the trials did not include patients with severe chronic diseases other than OSA, especially comorbid cardiovascular disease, which limits the interpretation of safety data. Details of BP assessment were not supplied in all publications, but office BP was measured in all publications providing detailed methods. Benefit-risk was based on a compensatory model for efficacy and safety but is open to challenge with ponderation. However, a repeated analysis based on the ratio (data not shown) provided evidence of the same order of benefit-risk, showing that there was no difference between the treatments but the same order of benefit-risk.

Recent studies have demonstrated the long-term efficacy and safety of solriamfetol^56^^,^^68^ and pitolisant.^69^ The safety profile of modafinil in a real-world setting across a range of prescribing indications was reported in a large questionnaire-based study of prescribing general practitioners in England. Most AEs had been reported previously, but adverse drug reactions (ADRs) in individual patients included cardiac and psychiatric events, especially at a higher modafinil dose.^70^

Current clinical practice for OSA therapy differs in Europe and the USA due to regulatory requirements. Selection of medication depends on physician and patient preferences, and comorbidities, notably cardiovascular. Further studies are required, notably longer-term RCTs which prioritise head-to-head comparisons to support the results of existing meta-analyses. In addition, robust real-world evidence is needed for authorised drugs in terms of their adherence, effectiveness and safety and especially in patients who are non-adherent for primary OSA therapy.

This network meta-analysis analysed the largest number of trials (20) of OSA to date and included only data from authorised drugs and comparators (mainly placebo). The study provided risk ratios and/or Z-scores for: efficacy by combining subjective and objective outcomes, QoL, overall and cardiovascular safety (blood pressure and heart rate) and benefit-risk, as well as univariate efficacy and safety variables. Finally, risk-utility analysis showed superiority of pitolisant compared with solriamfetol and modafinil which might aid clinical decision making. Longer-term RCTs which prioritise head-to-head comparisons of these medications are needed.

## Contributors

Philippe Lehert, Jean-Louis Pépin, Yves Dauvilliersand Raoua Ben Messaoud contributed to the conception, design, acquisition of data, analysis and interpretation of the data, participated in drafting, reviewing the manuscript and approved its submission. Jean-Louis Pépin and Philippe Lehert verified the underlying data.

Marie-Joyeux Faure, Jerryll Asin, Ferran Barbé, Maria Bonsignore, Winfried Randerath, Johan Verbraecken, Sonya Craig and Christian Caussé contributed to reviewing the manuscript and approved its submission.

All authors read and approved the final version of the manuscript.

## Data sharing statement

Data sharing is not applicable to this article because no datasets were generated or analysed during the current study. All data analysed during this study were extracted by published sources listed and fully referenced.

## Declaration of interests

This analysis has been sponsored by Bioprojet Pharma.

Jean-Louis Pépin has received grants or contracts from the National Research Agency, and lecture fees and travel grants from RESMED, SEFAM and Bioprojet.

Jerryll Asin received support from Bioprojet for attending meetings and/or travel; received grants or contracts (paid to his institute) from Philips, Somnomed and Zoll Respicardia; consulting fees (paid to his institute) from Zoll Respicardia; participation on a Data Safety Monitoring Board or Advisory Board (paid to his institute) from Zoll Respicardia; member of the Dutch Association of Sleep Medicine (no payment).

Ferran Barbé received support from Bioprojet for attending meetings and/or travel; received grants or contracts for sleep research from Instituto de Salud Carlos III.

Maria Bonsignore received payment or honoraria for lectures, presentations, speakers bureaus, manuscript writing or educational events from Bioprojet and Takeda; support for attending meetings and/or travel from Bioprojet; participation on a Data Safety Monitoring Board or Advisory Board for Bioprojet.

Winfried Randerath received study funding from Bioprojet; payment or honoraria for lectures, presentations, speakers bureaus, manuscript writing or educational events from Heinen & Löwenstein, Habel Medizintechnik, Jazz Pharmaceuticals, Inspire, Philips Respironics and Bioprojet; support for attending meetings and/or travel from Heinen & Löwenstein, Habel Medizintechnik, Jazz Pharmaceuticals, Philips Respironics and Bioprojet; personal fees for participation on a Data Safety Monitoring Board or Advisory Board for Bioprojet, Jazz Pharmaceuticals and Procter & Gamble; unpaid roles with the European Respiratory Society Head Assembly 4, Sleep Disordered Breathing (until September 2023), Guidelines Director elect 2024 and the German Respiratory Society, Secretary General (until March 2024), authorised member since March 2024.

Johan Verbraecken received study funding from Bioprojet; support for teaching courses (paid to his institute) from Air Liquide, Bioprojet, Inspire Medical Systems, Löwenstein Medical, Medidis, Mediq Tefa, Micromed OSG, Philips, ProSomnus, ResMed, Sefam, SomnoMed, SOS Oxygène, Tilman, Total Care, Vivisol, and Zoll Itamar outside the submitted work; royalties or licenses (paid to his institute) from Epilog; consulting fees (paid to his institute) from Desitin and Epilog; payment of honoraria for lectures, presentations, speakers bureaus, manuscript writing or educational events (paid to his institute) from Atos Medical, Idorsia, Inspire Medical Systems; support for attending meetings and/or travel from Bioprojet; past-President (since 2020) of the Belgian Association for Sleep Research and Sleep Medicine.

Yves Dauvilliers received payment or honoraria for lectures, presentations, speakers bureaus, manuscript writing or educationa events from Jass Pharmaceuticals, Bioprojet, Takeda, UCB, Orexia, Idorsia and Avadel; support for attending meetings and/or travel from Jazz Pharmaceuticals, Bioprojet and Avadel; participation on a Data Safety Monitoring Board or Advisory Board for Idorsia.

Raoua Ben Messaoud, Marie Joyeux-Faure and Sonya Craig have no declaration of interest.
