# Supplementary material for: Comparative efficacy, safety and benefit/risk of alerting agents for excessive daytime sleepiness in patients with obstructive sleep apnoea: a network meta-analysis
Source: eClinicalMedicine. 2024 Sep 19;76:102843. doi: 10.1016/j.eclinm.2024.102843 (PMC11437752; doi:10.1016/j.eclinm.2024.102843)
Supplement: Supplementary material [file mmc1.docx]

**Comparative efficacy, safety and benefit/risk of alerting agents for excessive daytime sleepiness in patients with obstructive sleep apnoea: A network meta-analysis**

**Supplementary Tables and Figures**

**Supplementary Table 1 Risk of bias**

| Study | Bias from randomisation | Bias due to deviations from the intended interventions | Bias due to missing outcome data | Bias in measurement of outcomes | Bias in selection of reported results | Bias due to period and carryover effects |
| --- | --- | --- | --- | --- | --- | --- |
| Bittencourt et al. 2008 | 2 | 1 | 1 | 1 | 1 | 0 |
| Black et al. 2005 | 1 | 1 | 1 | 1 | 1 | 0 |
| Chapman et al. 2014 | 1 | 1 | 1 | 1 | 1 | 0 |
| Dauvilliers et al. 2020 | 1 | 1 | 1 | 1 | 1 | 0 |
| Dinges et al. 2003 | 2 | 1 | 1 | 1 | 1 | 0 |
| Greve et al. 2014 | 2 | 1 | 1 | 1 | 1 | 0 |
| Herring et al. 2013 | 1 | 1 | 2 | 1 | 1 | 1 |
| Hirshkowitz et al. 2007 | 1 | 1 | 1 | 1 | 1 | 0 |
| Inoue et al. 2013 | 1 | 1 | 1 | 1 | 1 | 0 |
| Inoue et al. 2016 | 1 | 1 | 1 | 1 | 1 | 0 |
| Kingshott et al. 2001 | 2 | 1 | 2 | 1 | 1 | 1 |
| Krystal et al. 2010 | 2 | 1 | 2 | 1 | 1 | 0 |
| Pack et al. 2001 | 1 | 1 | 1 | 1 | 1 | 0 |
| Pépin et al. 2021 | 1 | 1 | 1 | 1 | 1 | 0 |
| Roth et al. 2006 | 2 | 1 | 2 | 1 | 1 | 0 |
| Schweitzer et al. 2019 | 1 | 1 | 1 | 1 | 1 | 0 |
| Strollo et al. 2019 | 3 | 1 | 1 | 1 | 1 | 0 |
| Weaver et al. 2009 | 1 | 1 | 1 | 1 | 1 | 0 |
| Weaver et al. 2020 | 1 | 1 | 1 | 1 | 1 | 0 |
| Williams et al. 2010 | 1 | 1 | 1 | 1 | 1 | 0 |

Bias was evaluated with the Cochrane, RoB 2 tool: 0 = not applicable; 1 = low risk of bias; 2 = probable low risk of bias, 3= high risk of bias

**Supplementary Methods**

**Data analysis: calculations and transformations**

For continuous endpoints, the mean ± standard deviation (SD) was most common parameter reported by studies. Pre-determined transformations were the conversion of median and quartiles into mean and SD, assuming an overall approximation to a normal distribution.

In general, missing data (mean, SD, or N) were imputed through an indirect estimate based on the observed value. In cases where it was not possible to find the SD either indirectly or directly, missing value imputation was first based on the SD found in the same study for the endpoint; otherwise, it was based on the mean value of the coefficient of variation for the endpoint across all studies. Least squares (LS)-mean or final or mean change values were computed, assuming a correlation between final and baseline values of 0·5. Values provided as standard errors (SE) were converted to SD (= SE*√n), and non-reported SE or SD values were estimated through the lower limit (LL) of (1-α) confidence interval using the expression SD = (mean-LL)/z1-α/2, where z1-α/2 is the 1-α/2 percentile of the normal distribution.

The conversion of mean ± SD data into binary variables was performed by fixing a threshold of response determined by medium clinical efficacy (SD/2), and calculating the proportions of responders in each arm, based on a normal distribution. When only the odds ratio (OR) was provided, the conversion of OR into Standardized Mean Difference (SMD) was calculated using OR=SMD.π /√3 [Murad et al. 2019]. Mean SMD was calculated as effect size (ES): ES=logn(m1)* 551, and var(ES) = (ES -logn(s1)*·551)/1·96. For two groups the calculation was: ES=(m1-m2)/SD, and eval varES= (n1+n2)/(n1*n2)+ES^2 /(2*(n1+n2)). If only the SMD was provided, varES= 2/n1+ES^2 /(4*n1) was calculated.

Numerical endpoints were converted into a binary variable based on a cut-off value, above and below which the numerical variable was dichotomized. For each continuous variable, a cut-off defined as half the standard deviation (SD/2) was used corresponding to the empirical medium efficacy threshold [Cohen, 1988].

Binary endpoints (ratios or proportion p=f/n) were converted into continuous variables through the normal approximation of the binomial distribution. The SMD was calculated based on the estimated proportion, and calculated from the mean (m), m=f/n and the standard deviation, SD=√m(1-m).

In one study, the same endpoint was measured with different tools. These values were pooled and transformed into their SMDs. The SD was approximated based on the mean effect size (ESi) and standard error (SEi) of each endpoint, assuming that k SEi and the k(k-1)/2 covariance are similar. Thus, the mean Z-score is the mean of the SEi, and the resulting SE was calculated as: SE_mean_/k * (1+(k-1)r); if r=0 , SE of the mean= SE/k; while if r=1, SE=SE_mean_. If these values are considered as independent, SMD total=mean/n where n is the number of replicates; whereas if these data are fully correlated, then SMD total=mean. This calculation is based on the approximation of the variance matrix (most often unknown) by a compound symmetry matrix with the same SD in the diagonal, and a common value of the correlation coefficient ρ. For composite endpoints based on different endpoints (such as safety), a composite score was calculated based on the sum of their Z-scores. The correlation between these variables within the same study was calculated through the Mahalanobis metric. When the correlation matrix was not reported or estimable, we assumed the same approximation of a compound symmetry with a common correlation of ρ=0·5.

Risk ratios were calculated for comparing proportions of categorical or binary endpoints.

**Statistical modelling**

Network meta-analysis was performed using the Rücker and Schwarzer method, a frequentist approach where an Aitken model for non-inversible matrices is calculated by the Moore-Penrose pseudo-inverse technique.^51^

**Supplementary Figures**

**Supplementary** **Figure 1. Forest plot for Epworth Sleepiness Scale (ESS).** Effect sizes (95% CI) relative to placebo are shown.


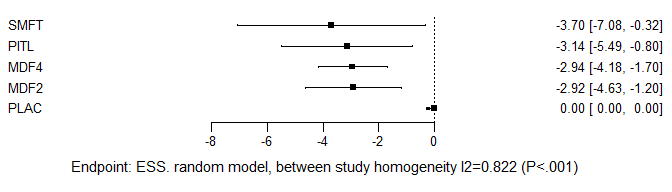


MDF2, modafinil 200 mg; MDF4, modafinil 400 mg; PITL, pitolisant; PLAC, placebo; SMFT, solriamfetol

**Supplementary** **Figure 2. Forest plot for Oxford Sleep Resistance Test (OSLER) / maintenance of wakefulness test (MWT).** Effect sizes (95% CI) relative to placebo are shown.


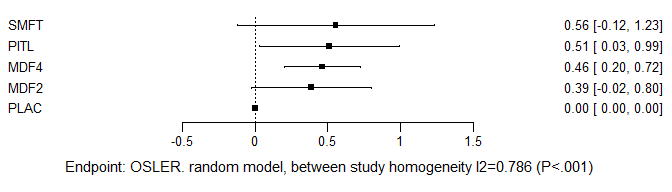


MDF2, modafinil 200 mg; MDF4, modafinil 400 mg; PITL, pitolisant; PLAC, placebo; SMFT, solriamfetol

**Supplementary** **Figure 3. Forest plot for quality of life (QoL).** Effect sizes (95% CI) relative to placebo are shown.


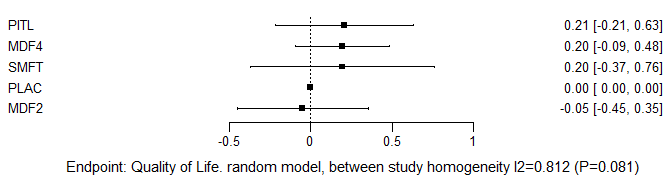


MDF2, modafinil 200 mg; MDF4, modafinil 400 mg; PITL, pitolisant; PLAC, placebo; SMFT, solriamfetol

**Supplementary Figure 4. Network diagram for safety**


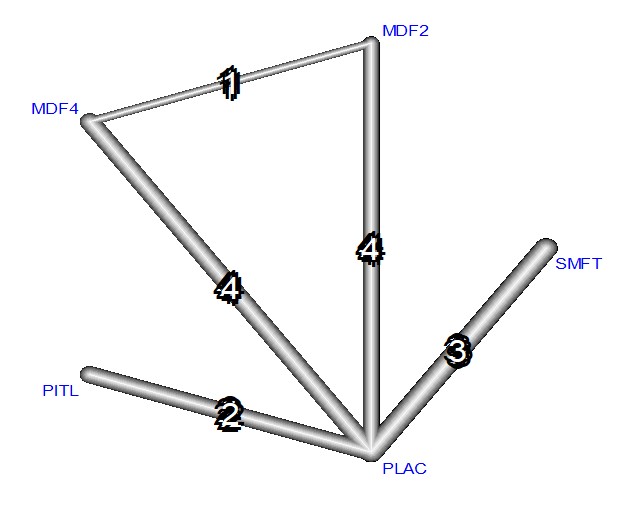


MDF2, modafinil 200 mg; MDF4, modafinil 400 mg; PITL, pitolisant; PLAC, placebo; SMFT, solriamfetol

The thickness of the lines corresponds to the number of trials included for each pairwise comparison. Numbers show the number of studies directly comparing treatments.

**Supplementary Figure 5. Forest plot for overall safety (Z-score).** Effect sizes (95% CI) relative to placebo are shown.


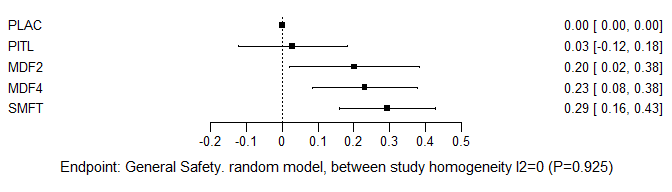


MDF2, modafinil 200 mg; MDF4, modafinil 400 mg; PITL, pitolisant; PLAC, placebo; SMFT, solriamfetol

**Supplementary** **Figure 6. Forest plot for cardiovascular safety (Z-score).** Effect sizes (95% CI) relative to placebo are shown.


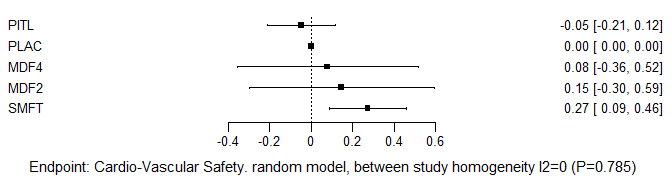


MDF2, modafinil 200 mg; MDF4, modafinil 400 mg; PITL, pitolisant; PLAC, placebo; SMFT, solriamfetol

**Supplementary** **Figure 7. Forest plot for Systolic Blood Pressure (SBP).** Effect sizes (95% CI) relative to placebo are shown.


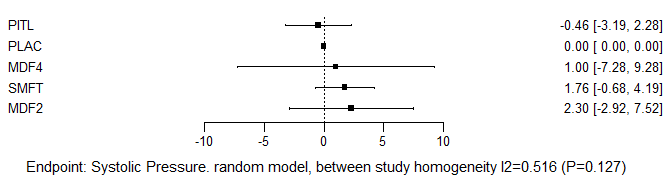


MDF2, modafinil 200 mg; MDF4, modafinil 400 mg; PITL, pitolisant; PLAC, placebo; SMFT, solriamfetol

**Supplementary** **Figure 8. Forest plot for Diastolic Blood Pressure (DBP).** Effect sizes (95% CI) relative to placebo are shown.


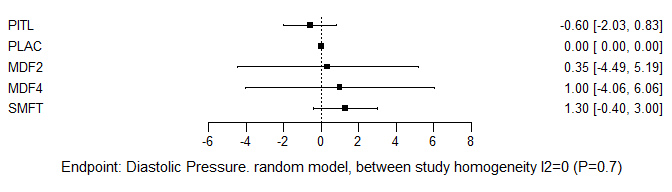


MDF2, modafinil 200 mg; MDF4, modafinil 400 mg; PITL, pitolisant; PLAC, placebo; SMFT, solriamfetol

**Supplementary** **Figure 9. Forest plot for Heart Rate (HR).** Effect sizes (95% CI) relative to placebo are shown.


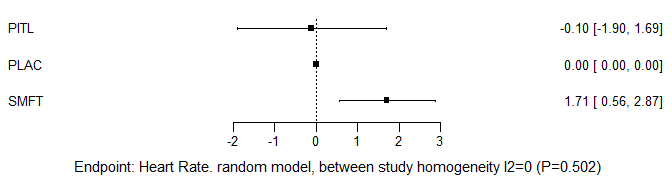


PITL, pitolisant; PLAC, placebo; SMFT, solriamfetol

**Supplementary** **Figure 10. Forest plot for all TEAEs.** Effect sizes (95% CI) relative to placebo are shown.


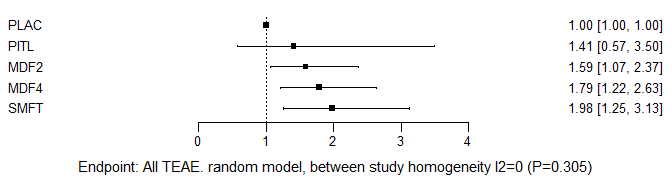


MDF2, modafinil 200 mg; MDF4, modafinil 400 mg; PITL, pitolisant; PLAC, placebo; SMFT, solriamfetol

**Supplementary** **Figure 11. Forest plot for headache.**  Effect sizes (95% CI) relative to placebo are shown.


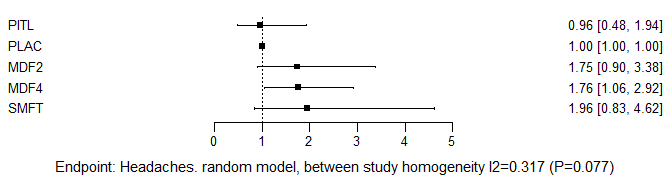


MDF2, modafinil 200 mg; MDF4, modafinil 400 mg; PITL, pitolisant; PLAC, placebo; SMFT, solriamfetol

**Supplementary** **Figure 12. Forest plot for TEAEs (except headache).**  Effect sizes (95% CI) relative to placebo are shown.


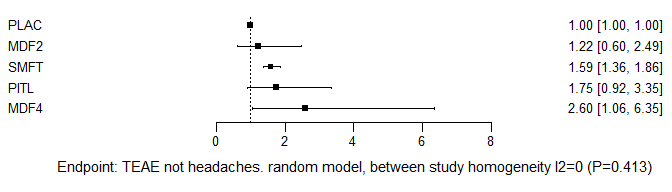


MDF2, modafinil 200 mg; MDF4, modafinil 400 mg; PITL, pitolisant; PLAC, placebo; SMFT, solriamfetol

**Supplementary** **Figure 13. Forest plot for Serious Adverse Events (SAEs).** Effect sizes (95% CI) relative to placebo are shown.


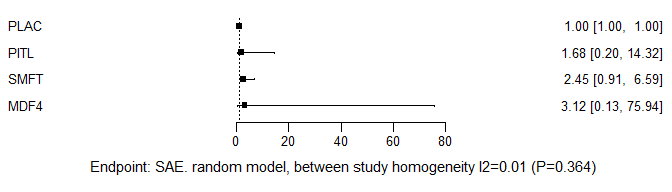


MDF4, modafinil 400 mg; PITL, pitolisant; PLAC, placebo; SMFT, solriamfetol
